# History and Future of KALIS: Towards Computer-assisted Decision Making in Prescriptive Medicine

**DOI:** 10.1515/jib-2019-0011

**Published:** 2019-05-30

**Authors:** Marcel Friedrichs, Alban Shoshi

**Affiliations:** Bielefeld University, Faculty of Technology, Bioinformatics/Medical Informatics Department, Bielefeld, Germany

**Keywords:** Drug-Related side effects and adverse reactions, computer-assisted decision making, polypharmacy, potentially inappropriate medication list, information systems

## Abstract

With an increasing older population in Germany and the need for polypharmacy to treat multimorbid patients computer-assisted decision making on an individual level is increasingly important to reduce prescription errors and adverse drug reactions. While current systems focus on guidelines and prescribing information, molecular information is equally important for explanation and discovery of drug-related problems. Based on the existing KALIS system and newer projects like PIMBase, a new concept for the KALIS-2 system is presented. Improvements to the modularisation of components enable future extension and greater maintainability. Interoperability with available electronic health records standards and protocols allows the integration and communication with existing workflows for healthcare professionals. Finally, new visualisation modes empower the user to explore and analyze the patient situation in an individual patient subgraph. For offline use and dialogue between patient and general practitioner, the results can be printed out using a new reporting tool. The adherence to findings from previous decision support systems and reasons for their failed adoption is an important task in the development of KALIS-2.

## 1 Introduction

Polypharmacy is defined as the concurrent use of multiple medications and often necessary for the treatment of multimorbid patients. With an increasing older population in Germany, prognosticated to be 22% of the population aged 65 and older in 2019 [1], the prevalence of multimorbidity is growing. Approximately 2.7 million BARMER insured people in Germany are suffering from five or more chronic diseases [2]. In addition, every fourth BARMER insured person aged 65 and older received at least one potentially inadequate medication (PIM) based on the PRISCUS list [2], [3]. If analyzed with more PIM lists like FORTA [4] or EU(7)-PIM [5] this result would likely be even higher. Further increasing the complexity of the prescription process is the growing number of available medications. The German Federal Institute for Drugs and Medical Devices (BfArM) reported for January 2019 approximately 103,000 medications on the German market. From these medications, 34,310 are freely available and 53,080 without a prescription [6]. Furthermore, polypharmacy increases the risk of drug-related problems such as medication errors, potentially inappropriate prescribing, and adverse drug reactions. No healthcare professional is likely able to review all of these possibilities without some form of a computer-assisted system.

## 2 Related Work

Many different decision support systems are available and in use by healthcare professions. They can be grouped into six types: dosing support, order facilitators, point-of-care alerts, relevant information displays, expert systems, and workflow support [7]. Each type represents a specific scenario and certain systems can be attributed to multiple types.

One of the major pharmacological databases in Germany is the commercial SCHOLZ database which includes a risk control system [8]. Multiple components are implemented including guidelines, drug interactions, adverse drug reactions, and PIMs based on the PRISCUS list.

Another commercial system is i:fox® which is available in multiple variants including a web-based implementation [9]. The system provides multiple components including drug interactions, contraindications, duplicate prescriptions, allergies, food interactions, and PIMs based on the PRISCUS list.

The European funded research project “Polypharmacy in chronic diseases: Reduction of Inappropriate Medication and Adverse drug events in elderly populations by electronic Decision Support (PRIMA-eDS)” focuses on the development and analysis of a decision support system for treatment of elderly populations. Implemented components include drug interactions guidelines, dose warnings, and PIMs based on European, American criteria and the EU(7)-PIM list [10].

All of these systems include the analysis of drug interactions for adverse drug reactions and PIMs. The commercial solutions on the German market focus primarily on the PRISCUS list, matching the current implementation of KALIS [11]. Only the PRIMA-eDS project utilizes multiple PIM lists. While the PRISCUS list is the primary list used in Germany the combination of multiple lists improves the coverage of knowledge about PIMs under different criteria and need to be considered in decision support systems. Focused on guidelines and professional prescribing information, molecular information from research mostly plays an inferior role in these systems. This information offer a deeper understanding and reasons for adverse drug reactions and their growing importance need to be considered.

## 3 History and Current Capabilities of KALIS

**Figure 1:**
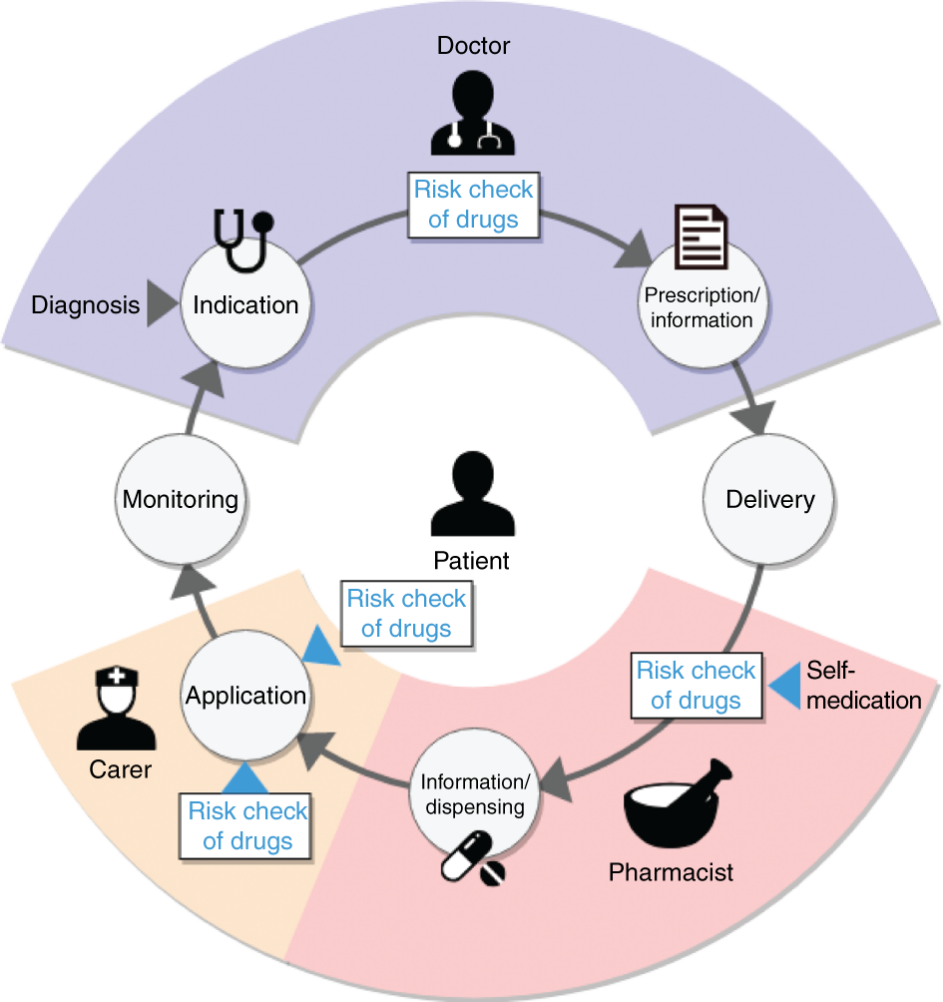
The prescription process can be split into distinct steps. Multiple points in the process-timeline exist where a risk-check of drugs is important to minimize medication errors and adverse drug reactions.

As illustrated in Figure 1, the prescription process can be split into several distinct steps and entities involved. Multiple time-points in this process exist where a risk-check of prescribed drugs on an individual level can be executed. The web-based system KALIS was developed to provide this capability in an intuitive way for healthcare professionals and patients alike [11]. KALIS is composed of multiple components for different scenarios that can be executed separately.

The main component of KALIS is the pharmacological risk-check. Based on information provided like prescribed medication or active agents, diseases or indications, side effects, and intolerances the system analyses for adverse drug reactions like drug interactions, contraindications, or double prescriptions. Additional conditions like pregnancy or food intolerances can be considered as well. In accordance with professional prescribing information the severity and full-text details for each result are provided to the user. A separate component is a check for potentially inappropriate medications based on the German PRISCUS list including 83 medications for patients aged 65 and older. The pharmacogenetic drug-drug interaction component analyses potential interactions based on Cytochrome P450 (CYP) metabolism of medications and allows for the input of specific CYP defects. While these components are based on professional prescribing information or expert panels, separate molecular risk-checks enable the analysis of drug side effects, drug interactions, and compound interactions based on scientific evidence. In contrast to the data-based analyses KALIS provides a component for patients with hypertension using medical guidelines. Based on patient specific values such as age, blood pressure, and creatinine levels KALIS provides medication recommendations to assist healthcare professionals in the prescription process.

## 4 Redesign and New Risk-Check Components for KALIS-2

The development of the KALIS-2 system involves a complete redesign to match new requirements and the technological changes that occurred in the web-development ecosystem. This section highlights several important changes in concepts and ideas for the future implementation of KALIS-2.

### 4.1 Input of Patient Data

At the center of the prescription process is the patient and all the medical parameters that may be assigned to the individual. Aside from standard information like age or weight, complex parameters such as prescribed drugs and dosages, diseases, side effects, and many more can be relevant for healthcare professionals to diagnose and treat patients safely. For using KALIS-2, the relevant information needs to be provided to the system in a fast and intuitive way. Feedback from healthcare professionals has shown that a critical role plays the time needed to input patient information and that interoperability with existing systems and electronic health records (EHR) would greatly decrease this limiting factor [10].

**Figure 2:**
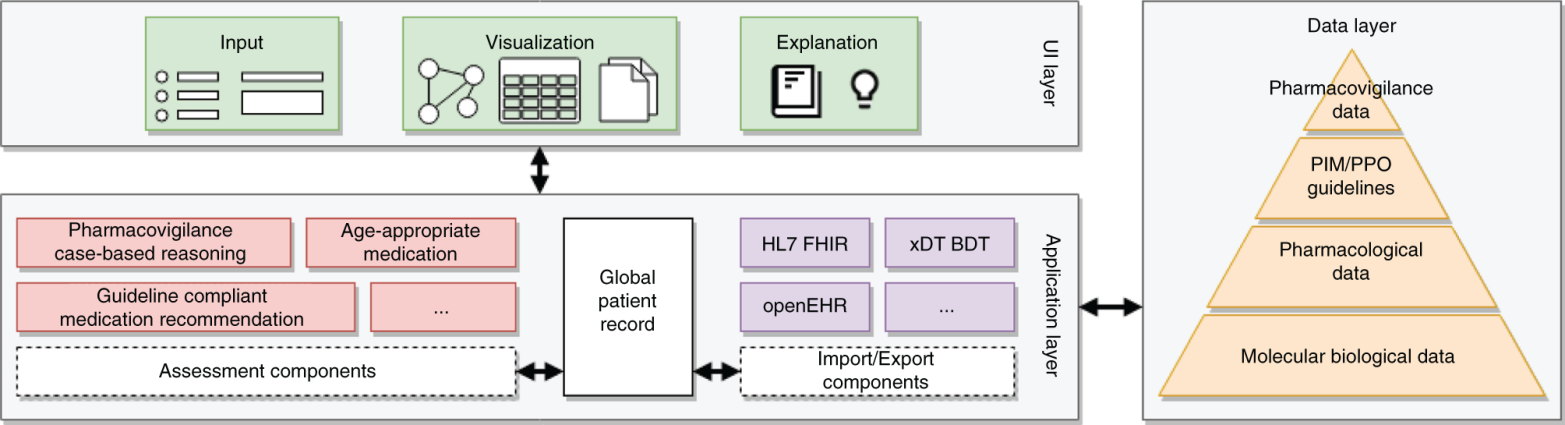
Concept of the KALIS-2 modularised system. The global patient record can be imported from and exported to different electronic health record (EHR) formats. The assessment components work on the patient record to analyse specific aspects like drug interactions. The results and explanations are provided to the user by a visualization component. All information are provided by a data layer ranging from large molecular up to patient specific and case-based data.

Different components of KALIS-2 require different patient parameters for analysis which may be exclusive to the component or overlap with others. To prevent duplicated input of information a global patient record is populated with the user input and used across all components as illustrated in Figure 2. Additionally, this enables the system to dynamically adjust the input forms depending on the activated components. If all information would be input in a single, large form the user could be easily overwhelmed and hesitant to use the system at all. Another advantage of the global patient record is the ability to represent an individual patient with this data structure and reuse it for analyses at a later point in time. Finally, to address the aforementioned feedback for interoperability, the global patient record can be imported from or exported to standardized EHR data formats. Possible candidate formats are Health Level-7 (HL7) FHIR [12], openEHR [13], and the German health care standard xDT BDT [14]. This import and export capability is modularised to be easily extended if necessary as illustrated in Figure 2. To ensure the security of patient data, several ideas are under consideration. Modern client-based web applications allow critical data to be stored on the client side only and are therefore not transmitted to the server. However, this approach makes it difficult to load already entered data on other devices. A data matrix on the printed reports storing all information may be a solution to this problem as done for the nationally standardized medication schedule in Germany. The alternative of persisted patient records on the server side requires strict encryption standards. This includes transport of data from client to server using SSL/TLS, hardware disk and database encryption. Additionally, EU general data protection regulations (GDPR) need to be applied.

### 4.2 Modularised Assessment Components

While the current KALIS version already follows the idea of modularisation in the web interface, the implementation is closely coupled and hard to distinguish. Therefore, the KALIS-2 system is implemented with modularisation at its core as illustrated in Figure 2. This allows for each component to be developed and maintained separately and for new components to be integrated in the future. Another advantage of this separation is the use of components that require free and/or licensed databases. While KALIS currently relies on licensed data and therefore is not openly available, the new system allows the distinction between openly available and closed components. Aside from the existing KALIS components described before, two active projects will be the focus for new components integrated into KALIS-2.

One of the KALIS components is a check for potentially inadequate medication (PIM) using the PRISCUS list including 83 drugs. Due to physiological and metabolic changes in elderly patients aged 65 and older certain drugs are considered potentially inappropriate with regards to their benefit-risk ratio. With the PRISCUS list being the most used list in Germany many different lists exist. Some are tailored to specific countries while others for example only consider patients in nursing homes or with certain indications. PIMBase [15] is the first publicly available iteration of a web-based system combining multiple lists and providing them in a digitally accessible manner. Using the combined lists in PIMBase already shows an increased coverage of drug classes and indications for PIMs. The integration of PIMBase as a component for KALIS-2 is a logical step replacing the PRISCUS component to further increase personalized risk-assessment for older patients with more PIM lists. Individual parameters like patients from different countries could thus be considered by using PIM lists tailored for patients of different origin. PIM entries with more detailed constraints, like patients with a history of certain diseases, can be selected if the patient provides these information. With new or updated PIM list revisions being published roughly every 2–5 years, this component can be extended in a short amount of time after a lists release. This is achieved using a unified, semi-automatic integration pipeline generating the PIMBase database [15].

Based on the current implementation of the hypertension medication assistant in KALIS, other patient specific conditions are integrated. An important example is the consideration of renal function, especially in elderly patients. In the case of renal failure, the addition of glomerular filtration rate (GFR) data allows for the analysis and avoidance of potential adverse drug reactions caused by a modified drug clearance.

GenCoNet is a graph database for the analysis of comorbidities by gene networks [16]. Information about drugs that may cause comorbidities or which genes are potential factors in comorbidities can be extracted. KALIS integrates only a limited number of biological molecular information like the CYP450 database. With the increasing number of molecular information available, the potential for medically relevant inferences based on molecular data also increases. Therefore, the integration of GenCoNet as a component for KALIS-2 provides new possible assessment opportunities in individual patient situations for further investigation. Other molecular information like pathways for drug metabolising and genetic factors from curated databases and scientific literature are implemented to enable detailed perspectives for the explanation of individual patient situations.

### 4.3 Result Visualization and Reporting

Due to the modularisation of KALIS-2, each component generates a separate set of analysis results that need to be provided to the user in a concise way with the option to access more detailed explanations if necessary. Two different modes of result presentation are provided, the interactive web interface and a reporting tool that is tailored for printing out the results.

**Figure 3:**
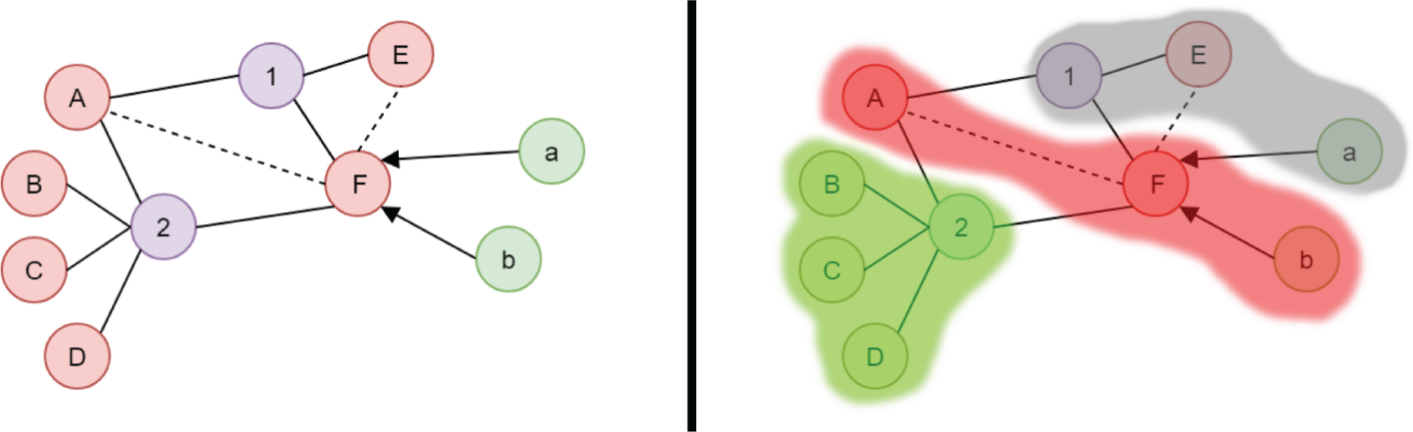
Simplified example of the whole medical graph (left) and the relevant patient subgraph (right). Drugs are labeled with upper case letters, diseases with numbers and potentially inappropriate medication (PIM) entries with lower case letters. The example patient is diagnosed with disease “2”. Information inside the gray area are therefore not relevant to his current situation. PIM entry “a” is not relevant because of additional conditions not met by the patient. Drug “A” and “F” are indicated for disease “2” but are known to interact and drug “F” is a PIM, indicated by the red area. Drugs indicated for disease “2” without known problems are highlighted with a green area.

Like the current implementation of KALIS, results are listed in the web interface in a structured format, like tables or lists and lead to detailed information through an intuitive user interface (UI) flow and dialogues. Certain result information like numerical scale values or classification classes are color coded or abbreviated with a legend. This allows the user to directly identify relevant information and to further investigate the results most relevant to the situation. In addition, a new concept of result representation is added. The complete medical and prescription process can be represented as a large graph with entities like drugs or diseases as nodes and relationships like indications, alternatives, adverse reactions and more. This complete graph is too big to survey at a glance and many parts are most likely not relevant to the current patients’ situation. KALIS-2 tries to identify the relevant information and builds a personalized patient subgraph, which can be reviewed and explored directly in the web interface. A simplified example for this graph is illustrated in Figure 3.

Reviewing and exploring the components results is intended to be a responsive and intuitive process in the web interface. However, for situations without internet or where no digital device is available to access the system the results can also be printed out using the KALIS-2 reporting tool. In contrast to the web interface hiding more detailed information behind UI flows, the printed version needs to include all relevant information at once. Starting the reporting tool opens a preview and separate interface for adjusting the output. For example, if certain results or detailed explanations are not relevant in this situation they can be excluded from the report. Additionally, different layouts can be selected to resemble a more patient or healthcare professional focused view. Finally, QR codes can be added where external information are linked like classification systems or other web-based information systems. This printed version of the results enables the patient for example to mark certain parts that are unclear or of interest and discuss them with their general practitioner (GP).

In addition to the visualization and reporting of results where the user has to infer meaning themself, an explanation component is provided to assist in the prescriptive decision making process. Many guidelines include algorithms or processes to adjust prescriptions based on certain patient information as implemented in KALIS for hypertension. Providing concise explanation and reasoning for the results KALIS-2 generates for specific patients is important to ensure trust in the system. Furthermore, it enables healthcare professionals to make more informed decisions on an individual patient level based on evidence such as laboratory measurements in the case of renal failure or hypertension.

## 5 Discussion

The development of the new KALIS-2 concept is an important step towards extensibility and maintainability. New results in research and modified medical guidelines demand the constant evaluation and adaption of computer-assisted decision support systems. However, the availability of such systems is not a guarantee for the adoption by healthcare professionals [17]. Many factors influence the adoption and need to be addressed in the development of new systems. The most important factor is the computer availability which has improved due to the ongoing digitisation of GP offices. In Germany 60% of GPs are reported to be using a computer system for medication safety [18]. Furthermore, the integration of new systems in existing infrastructure can be complicated and software or hardware problems may lead to frustration and non-use of the system [17]. This can be addressed with proper documentation and case based help tools. While patients using the system are likely interested in all provided information due to the relevance to their own health, healthcare professionals reported the possibility for desensitization to alerts if they appear intrusive, too information-dense, inconsistent in vocabulary or appear too frequent [7], [17]. These factors show the importance for precise and intuitive UI-flows in KALIS-2 that can be adopted in existing workflows and assist rather than interrupt healthcare professionals.

## 6 Conclusion

This concept for the KALIS-2 system represents the next logical steps for KALIS towards improving medical decision making in a personalized and computer-assisted way. The interoperability with health care standards for EHRs and license free components will help to make the system more accessible and adaptable for healthcare professionals and patients alike. Patients are given the opportunity to print out their results with the reporting tool and start a more informed discussion with their GP. The adoption of a broader spectrum of analysis components will help gain a more complete picture of the patients’ health situation and possible steps to improve the prescription process. Finally, the new modularisation will help keep the KALIS-2 system up to date and extensible for future extending projects.
